# Gluteal muscle damage leads to higher *in vivo* hip joint loads 3 months after total hip arthroplasty

**DOI:** 10.1371/journal.pone.0190626

**Published:** 2018-01-09

**Authors:** Philipp Damm, Jip Zonneveld, Sophie Brackertz, Florian Streitparth, Tobias Winkler

**Affiliations:** 1 Julius Wolff Institute, Charité, Universitätsmedizin Berlin, Berlin, Germany; 2 Klinik für Radiologie, Charité, Universitätsmedizin Berlin, Berlin, Germany; 3 Berlin Brandenburg Center for Regenerative Therapies, Charité, Universitätsmedizin Berlin, Berlin, Germany; 4 Center for Musculoskeletal Surgery, Charité, Universitätsmedizin Berlin, Berlin, Germany; University of Memphis, UNITED STATES

## Abstract

**Background:**

Total hip arthroplasty (THA) is in most cases improving patients´ life quality immediately after surgery. However, a closer look at these patients, especially with modern gait analysis methods, reveals also negative consequences due to the surgical approach related injury to the pelvic muscles. We hypothesized that this damage will have a negative impact on hip joint contact forces during activities of daily living (ADL).

**Methods:**

10 patients undergoing THA received an instrumented hip joint implant enabling real time *in vivo* measurements of hip joint loads using a direct lateral approach. Pre- and 3 months postoperative computed tomography (CT) scans were used for evaluation of the periarticular muscle status, using muscle volume, fat ratio and lean muscle volume as parameters. An analysis of in vivo hip contact forces was made 3 months after THA during ADL (walking, stair climbing, chair rising and sitting) and correlated with the morphology of the periarticular muscles.

**Results:**

We found a significant decrease of volume by 25% (-3 to -45, p = 0.005) and increase in fat ratio of the Gluteus Minimus (Gmin), resulting in a decrease in lean muscle volume of 28% (-48 to 0, p = 0.008). This was accompanied by an inverse development in the Tensor Fasciae Latae (TFL) resulting in a lean muscle volume increase of 34% (-2 to -102, p = 0.013). Changes in Gluteus Medius (Gmed) and Gluteus Maximus (Gmax) have not been observed in the short-term follow up. A decreased Gmin lean muscle volume was found to strongly correlate with high in vivo joint contact forces in all tested ADL.

**Conclusion:**

The decrease of Gmin volume can be seen as a direct effect of THA surgery, whereas the increase of TFL might compensate for loss of Gmin volume. Lean muscle volume and fat ratio were better predictors for joint contact forces than total muscle volume. These effects were most pronounced during sitting down and standing up due to the higher demand on the gluteal muscles during these activities.

## Introduction

In Germany, more than 200 000 total hip arthroplasties (THA) are performed per year [1]. THA can increase quality of life and mobility [2], yet it can also lead to negative consequences. These negative effects can be related to the surgical approach and include atrophy, fatty degeneration and loss of function in the hip surrounding muscles [3,4], which in return influences general functionality and mobility of the patient. Both, atrophy and fatty degeneration, have been shown to negatively influence the postoperative outcome [5–7]. In studies investigating muscle or tendon repair of the shoulder and hip, fatty degeneration has been shown to negatively impact the functional outcome and muscle strength [5]. An increase in the fatty degeneration of the gluteal muscles is often associated with impaired walking patterns and other ADL such as chair rising [8]. An abnormal gait pattern, such as the Trendelenburg gait, can cause higher in vivo joint contact forces [9,10] which can be a potential harm to implant longevity [11].

The gluteal muscles, along with the TFL, play a central role in stabilizing the hip joint in most ADL [12–18], a damage can thus entail serious negative consequences [19]. Whereas joint contact forces are influenced by muscle status, implantation angle and lever arm of contributing muscles [16,20,21], the periarticular muscles of the hip determine up to 95% of the joint contact forces during walking [14].

Using the direct lateral approach (DLA), a widely performed THA approach [22–24], gluteal muscle damage is inevitable for good exposure of the joint [25] with the aforementioned patient risks, including abnormal postoperative gait patterns. Prior research has indicated the possibility, that a damage to periarticular muscles lead to increased joint contact forces [16,26], however, to our best knowledge, there are no in vivo studies that correlate the hip muscle status with joint contact forces. Most analyses of the impact of muscle status on joint contact forces address gait patterns or ground reaction forces and can only extrapolate on joint forces [27]. This study aims at investigating the influence of the periarticular hip muscle status on the joint contact forces in THA patients 3 months after surgery. Based on previous research we hypothesized that an impaired gluteal muscle status will correlate with higher in vivo joint contact forces in the hip.

## Methods

### Subjects and study design

We retrospectively analyzed individual in vivo measured hip joint loading and muscle status. All patients were operated using the direct lateral approach (n = 10, mean age 57.3 years, 50 to 68, female: n = 2, Table 1) and received an instrumented hip implant ([28]). The study was approved by the Charité Ethics committee (EA2/057/09) and registered at the ‘German Clinical Trials Register’ (DRKS00000563). All patients gave written informed consent prior to participating in this study.

**Table 1:** Demographic characteristics of patient collective 3 months after THA.

Subject	H1L	H2R	H3L	H4L	H5L	H6R	H7R	H8L	H9L	H10R
Age [years]	56	61	60	50	63	68	53	55	54	53
BMI [kg/m^2^]	24	27	31	25	31	27	28	25	34	37
Sex [f / m]	m	m	m	m	f	m	m	m	m	f

BMI = Body Mass Index, R = right operated side, L = left operated side.

### Computed tomography (CT)

All patients were scanned using helical CT (Toshiba Aquilion ONE, V4.61GR004, Tokyo, Japan; 120kV, 200mAs, FOV 40cm). Original CT scans were reconstructed to 5mm files (GE Medical systems, software version vxtl_12.3–2.86, volume viewer, smooth 1 filter). CT scans were obtained one day prior to and three months after surgery.

### Image analysis

Using dedicated software (Osirix Imaging Software, Geneva Switzerland; Amira Visage Imaging, Berlin, Germany) for CT analysis, muscle slices with 5 mm distance were manually outlined and intermediate surface completed using interpolation. To control for variation in patient body heights, volumes were measured between the anatomic landmarks of the fourth lumbar vertebrae (L4) and the lesser trochanter (LT). Muscular fatty degeneration was assessed using a standardized Hounsfield Unit (HU)–based approach [8,29]. For the gluteal muscles, three consecutive slices were selected for analysis starting 3 cm cranial of the greater trochanter (GT) as a reproducible anatomic landmark. The TFL was measured in three consecutive slices inferior to the superior anatomical aspect of the GT.

### In vivo load measurement

In vivo joint contact loads were collected using instrumented hip implants, a detailed description of the prostheses has been previously published by Damm et al. [28]. The following ADL were included in our protocol: level walking (self-chosen speed by the patients), stair climbing without support, chair sitting and rising. The resultant force (F_res_) was calculated from the force vectors F_x_, F_y_ and F_z._ which respectively act in the lateral, anterior and superior direction of axis of the femur. F_res_ are reported in Newton (N) and percentage body weight (%BW). Periarticular muscles are active during specific time points in the activity cycle. Hence, each periarticular muscle influences the movement and actions during gait cycle differently [12–18]. For the appropriate correlations between muscle status and joint contact forces we searched during which peak each periarticular muscle is active in the ADL activities (see Table 2). This enabled us to identify which muscle damage could have an impact on joint contact forces.

**Table 2:** Activity pattern of the ipsilateral hip muscles at defined time points of the joint loads (shown in Fig 1).

ADL	Gluteus minimus	Gluteus medius	Gluteus maximus	TFL
Walking 1 Peak	+	+	+	+
Walking 2 Peak	+	+	-	-
Stairs up 1 Peak	+	+	+	+
Stairs Down 1 Peak	+	+	+	+
Sit Down Max	+	+	+	+
Stand up Max	+	+	+	+

active = +, not active = -

### Statistical methods

The Mann-Whitney U test was used for testing for inter-individual, and Wilcoxon’s test for intra-individual differences in pre- and postoperative muscle volume and fat ratio. Correlations between volume and fat ratio of a muscle and vivo joint loads were analyzed with the use of the two tailed Spearman rank test. Statistical analyses were performed using IBM SPSS (SPSS, 2013), *p*<0.05 was considered significant.

## Results

### Muscle status

Three months postoperative changes, compared to preoperative values, of volumes and fat ratio are displayed in Table 3. Total volume of the Gmin muscle decreased by 25% (-45 to -3, *p* = 0.005), which was accompanied by a non-significant increase of total TFL volume by 14% (-10 to 56, *p* = 0.059).

**Table 3:** Mean postoperative volume of the ipsilateral side in [%].

Muscle		Gmin	Gmed	Gmax	TFL
Total muscle volume		-25 (15.3)**	6 (12.5)	-2 (9.8)	14 (21.9)
Lean muscle volume		-28 (14.4)**	3 (15.1)	-9 (21.8)	34 (31.2)**
Fat volume		-2 (42.7)	48 (112.0)	31 (94.3)	-52 (36.8)*
Fat ratio		32(56.8)	36 (96.4)	31 (80.8)	-57 (32.1)*

SD = standard deviation, significance level

** = 0.01

* = 0.05

Lean volume of the Gmin muscle decreased by 28% (-48 to 0, *p* = 0.008), Gmed and Gmax showed minor, insignificant changes and TFL lean volume increased by 34% (-2 to 102, *p* = 0.013).

The indicator of fatty degeneration, fat ratio, was found to be increased in all gluteal muscles, but lacked significance (Gmin p = 0.169, Gmed p = 0.445, Gmax p = 0.475). The TFL showed a substantial decrease in fat ratio of 57% (-98 to -11, *p* = 0.017).

### Joint contact forces

**Fig 1:**
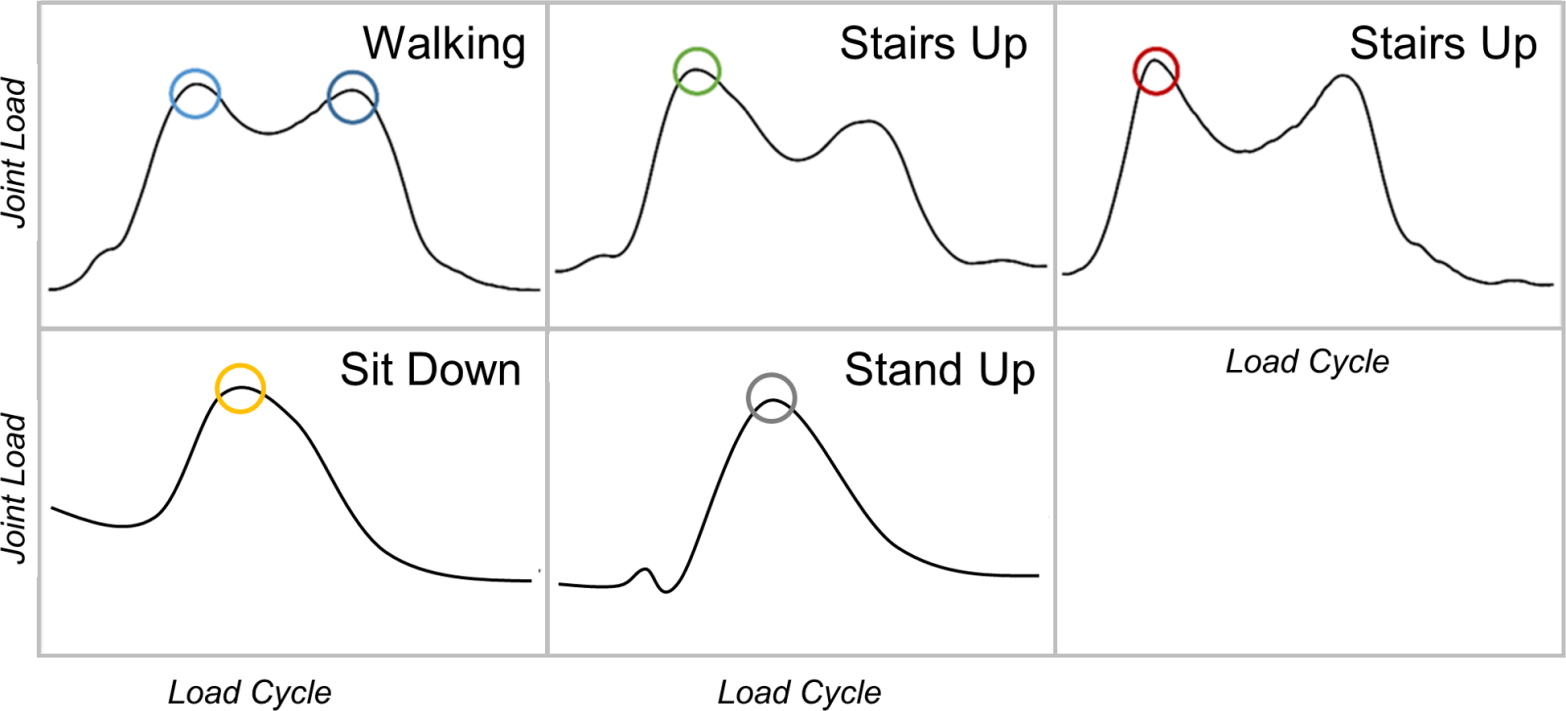
Average load patterns of in vivo measured hip joint contact forces during different ADL. Indicated are investigated peak values in all patients.

Inter-individual differences of peak forces were relatively small in walking and stair climbing, but showed large differences between patients during sitting down and standing up (Fig 1 and Table 4). The overall highest loads were observed during stair descend, with average peak forces of 281%BW (198–418) at the 1^st^ peak. The lowest peak forces were measured during sitting down, with an average of 169%BW (109–277). Patient H5 reached the highest single peak forces during almost every exercise, which was most pronounced in stair negotiation (336%BW in ascent; 418%BW in descent).

**Table 4:** Individual contact forces and load cycles during sit down / stand up at 3M after THA in [%BW (N)].

Patient		H1L	H2R	H3L	H4L	H5L	H6R	H7R	H8L	H9L	H10R	Average (SD)
3M	Walking	225	230	209	249	294	246	293	301	275	228	255 (33)
	1 P	[1679]	[1808]	[1841]	[1950]	[1530]	[2044]	[2634]	[2346]	[3044]	[2197]	[2209(429)]
	Walking	221	250	217	234	270	224	228	288	217	235	238 (24)
	2 P	[1666]	[1959]	[1915]	[1829]	[2326]	[1860]	[2045]	[2241]	[2408]	[2260]	[2051(246)]
	Stairs Up	200	252	212	303	336	254	266	304	283	263	267 (42)
	1 P	[1509]	[1976]	[1871]	[2373]	[2896]	[2111]	[2395]	[2372]	[3135]	[2535]	[2317(480)]
	Stairs Up	172	224	204	190	324	237	222	291	200	268	233 (48)
	2 P	[1294]	[1756]	[1799]	[1491]	[2794]	[1970]	[1994]	[2269]	[2218]	[2386]	[1997(442)]
	Stairs Down	217	246	198	325	418	298	253	299	273	282	281 (62)
	1 P	[1635]	[1933]	[1745]	[2548]	[3604]	[2477]	[2272]	[2328]	[3029]	[2722]	[2429(598)]
	Stairs Down	192	233	250	249	388	243	233	300	218	253	256 (54)
	2 P	[1450]	[1450]	[2202]	[1947]	[3344]	[2020]	[2099]	[2338]	[2394]	[2437]	[2206(498)]
	Sit Down	109	154	116	145	277	NA	NA	NA	NA	212	169 (64)
	Max	[819]	[1208]	[1004]	[1158]	[2387]					[2044]	[819(628)]
	Stand Up	103	151	130	192	355	NA	NA	NA	NA	238	195 (92)
	Max	[776]	[1183]	[1127]	[1158]	[2387]					[2291]	[1487(677)]

SD = standard deviation, NA = not available

### Correlation between muscle status and joint contact forces

Total muscle volume showed, beside Gmed volume (r_s_ = 0.67, p = 0.003) with walking 1^st^ Peak, no significant correlations with joint contact forces.

However, a decreased lean muscle volume of the Gmin showed strong correlations with higher contact forces in all exercises (r_s_ = -0.67 - -0.94, p = 0.035–0.005). Lean muscle volume of the other hip muscles did not correlate significantly with the joint contact forces (see Table 5). A lower lean volume of the GMax, however, showed a trend to correlate with higher contact forces in sitting down (r_s_ = -0.71, p = 0.11).

**Table 5:** Correlation of ipsilateral lean volume in [cm^3^] and in vivo joint contact forces in [N] at 3 months.

Ipsilateral		Gluteus Minimus	Gluteus Medius	Gluteus Maximus	TFL
3M	Walking 1 P	r_s_ = -0.67* (0.035)	r_s_ = 0.24 (0.51)	r_s_ = 0.30 (0.41)	r_s_ = 0.47 (0.17)
	Walking 2 P	r_s_ = -0.75** (0.013)	r_s_ = -0.08 (0.83)	r_s_ = -0.02 (0.96)	r_s_ = 0.41 (0.24)
	Stairs Up 1 P	r_s_ = -0.82** (0.004)	r_s_ = 0.02 (0.96)	r_s_ = 0.07 (0.86)	r_s_ = 0.10 (0.78)
	Stairs Down 1 P	r_s_ = -0.61 (0.06)	r_s_ = -0.18 (0.63)	r_s_ = -0.24 (0.51)	r_s_ = -0.04 (0.91)
	Sit Down Max	r_s_ = -0.94** (0.005)	r_s_ = -0.66 (0.16)	r_s_ = -0.71 (0.11)	r_s_ = -0.14 (0.79)
	Stand Up Max	r_s_ = -0.89** (0.019)	r_s_ = -0.49 (0.33)	r_s_ = -0.60 (0.21)	r_s_ = 0.09 (0.87)

r_s_ (p-values) calculated using Spearman’s rank correlation, significance level

** = 0.01

* = 0.05; P = Peak

Fat ratio showed strong correlations between all gluteal muscles and forces during sitting down (Gmin r_s_ = 0.93, p = 0.008; Gmed r_s_ = 0.94, p = 0.005; Gmax r_s_ = 0.84, p = 0.036) and standing up (Gmin r_s_ = 0.84, p = 0.036; Gmed r_s_ = 0.89, p = 0.019; Gmax r_s_ = 0.75, p = 0.08). The fat ratio of the Gmed, Gmax and TFL were further shown to correlate with 2^nd^ peak walking joint contact forces (r_s_ = 0.65–0.73, p = 0.042–0.018) (Table 6).

**Table 6:** Correlation of ipsilateral fat ratio in [%] and in vivo joint contact forces in [%BW] at 3 months.

Ipsilateral		Gluteus Minimus	Gluteus Medius	Gluteus Maximus	TFL
3M	Walking 1 P	r_s_ = 0.03 (0.93)	r_s_ = 0.31 (0.39)	r_s_ = 0.14 (0.70)	r_s_ = 0.09 (0.80)
	Walking 2 P	r_s_ = 0.20 (0.58)	r_s_ = 0.71* (0.022)	r_s_ = 0.73* (0.018)	r_s_ = 0.65* (0.042)
	Stairs Up 1 P	r_s_ = 0.20 (0.59)	r_s_ = 0.32 (0.37)	r_s_ = 0.18 (0.63)	r_s_ = 0.21 (0.58)
	Stairs Down 1 P	r_s_ = 0.05 (0.89)	r_s_ = 0.43 (0.21)	r_s_ = 0.39 (0.27)	r_s_ = 0.33 (0.34)
	Sit Down Max	r_s_ = 0.93** (0.008)	r_s_ = 0.94** (0.005)	r_s_ = 0.84* (0.036)	r_s_ = 0.14 (0.79)
	Stand Up Max	r_s_ = 0.84* (0.036)	r_s_ = 0.89* (0.019)	r_s_ = 0.75 (0.08)	r_s_ = 0.26 (0.62)

r_s_ (p-values) calculated using Spearman’s rank correlation, significance level

** = 0.01

* = 0.05; P = Peak

## Discussion

This study aimed at investigating the relationship between hip muscle status and in vivo hip joint contact forces after THA. To our knowledge, this is the first study worldwide comparing the individual hip muscle status and the *in vivo* measured joint contact forces. Our results generally support our hypothesis that an impaired muscle status, determined by muscle atrophy and fatty degeneration, corresponds with higher in vivo joint contact forces. In contrast to our expectations, total muscle volume was not related, but lean volume and fat ratio of periarticular muscles showed stronger correlations with an increase of in vivo joint contact forces. This effect was most pronounced for the Gmin muscle. Out of all analyzed activities, fat ratio and joint contact forces were best correlated during sitting down and standing up in all gluteal muscles. The TFL failed to show any relation between the muscle status and joint contact forces, despite its notable changes from pre- to postoperative.

The peak forces measured in our study are comparable to those described in the literature and are a good representation of normal joint contact forces in daily life ^9,26^. Three months after THA we found a decrease in the ipsilateral Gmin muscle volume, which has previously been described in the literature [30–33]. This decrease reflects direct muscle damage inherent to the lateral approach, a partial or complete detachment of the Gmin muscle playing a role in the mechanism of injury [34]. However, the contralateral decrease in lean volume indicates that not only direct damage can result in a loss of this important hip stabilizer.

The hypertrophy of ipsilateral TFL was a finding, that also confirms previous analyses of patients receiving THA via a direct lateral approach, and has been ascribed to decreased gluteal muscle function due to damage associated with surgery[25,35,36]. The TFL is, together with Gmin and Gmed, part of the hip abductor muscles and involved in stabilizing the hip joint during various movements[36]. Atrophy of one part of the abductor group can be compensated by hypertrophy of another. Furthermore, increased loading post surgery can also be a cause of the TFL hypertrophy[35]. TFL hypertrophy is most likely a compensatory mechanism for the loss of gluteus minimus.

We hypothesized that impaired muscle function would correspond with higher in vivo joint contact forces. For a controlled joint function, intact muscles are of utmost importance–with peaks in joint contact forces being one possible consequence of an uncontrolled contraction pattern due to muscle damage [37,38].

We initially considered the total muscle volume as sufficiently descriptive parameter for muscle status. Contrary to our considerations, total muscle volume and joint contact forces were only weakly correlated, underlining the need of qualitative parameters of muscle substance rather than un-corrected parameters when describing functional correlations. Gmin lean muscle volume, which is the volume of the muscle after deduction of the intramuscular fat, was strongly inversely correlated with joint contact forces. This suggests that lean muscle volume, and not total muscle volume, is a better predictor of muscle function, as lean muscle volume consists only of functional and contractile muscle tissue.

Besides muscle atrophy, we hypothesized that a higher fat ratio would also correspond with higher joint contact forces. Higher gluteal fat ratio can lead to an impaired gait cycle[8] and impaired gait cycles correspond with higher in vivo joint contact forces [9,10,39]. In the gluteal muscles, we observed a positive trend with higher fat ratios corresponding with higher joint contact forces. High fat ratio has been described to be a predictor of clinical surrogate of muscle function, possibly by the increasing stiffness of the muscle and thereby decreasing force[8] [40], apart from the accompanying loss of contractile muscle substance. The strongest correlation we observed was during standing up and sitting down. Although the joint contact forces are higher during stair walking, the physical demand on the gluteal muscles is higher during sitting down[41,42] where they act as hip stabilizers and extensors.

Other factors, besides muscle damage, can cause joint contact forces to increase. The contribution of other muscles that take over the function of impaired muscles could have influenced the joint contact forces. Bergmann et al.[26] described that various muscles with different angles at the hip joint may cause the increase in joint contact forces [9]. Since muscles make up 50–90% of the in vivo acting joint loading, the changes in muscle status will be of great importance.

Although Gmin is presumed to have less influence on the body balance than Gmed [14,18], we observed that loss of Gmin muscle resulted in higher forces in the hip joint. The significant correlation of Gmin lean muscle volume and fat ratio with higher contact forces might indicate that the Gmin plays a more important role in stabilizing the hip during sitting down and standing up than expected [14,18]. Literature about Gmin activation and precise function is scarce. To our best knowledge, only one study investigated Gmin separately using electromyography [18] and its action during walking. Other studies analyzed the combination of Gmin and Gmed [31].

Little is known about the exact contribution of the Gmin in daily activities, so its activation might be different than that of the Gmed during stair climbing or descending[12,14,18]. The relation we found during walking and stair climbing might give an insight of the function of Gmin, describing its important role during these activities.

### Limitations of our study design

This study has several limitations. First, our study cohort consists of ten patients, which is too small to draw firm conclusions. Second, in vivo joint loading information from the preoperative situation was not available. It is possible that the loads stayed the same while muscle status changed. In our study, we focused on a short-term follow up after THA. We have reason to believe, that within the first 3 postoperative months period the most pronounced changes in the periarticular musculature take place, since muscle regeneration will to the largest extent be completed within this time frame. Nevertheless, future research will also analyze long-term data, following the question if a post-injury reorganization of the periarticular musculature leads to changes in the relation of muscle status and contact forces. Future studies should also assess clinical outcome data, which we did not do in this study. This might give a better insight in the clinical consequences of the elevation of hip contact forces due to muscle damage. Finally, we only presented data for the ipsilateral side. Although contralateral gluteal muscles also contribute to ipsilateral joint contact force, they only contribute up to a maximum of 15% of joint contact forces [12].

## Conclusion

These results contribute to knowledge of understanding the influence of muscle damage on joint contact loads. The hypertrophy of the TFL showed that a compensation mechanism exists for the hip abductor group after initial gluteal muscle damage. The lack of correlation of total muscle volume and the evident correlation of fat ratio and lean muscle volume shows, that, when assessing post-THA recovery, no reliable assumptions can be made on the total muscle volume regarding joint contact forces.

## References

[1] OECD. Health at a Glance 2013. In: OECD Indicators OECD Publishing [Internet]. 2013. Available: http://dx.doi.org/10.1787/health_glace-2013-en

[2] Naal FD, Impellizzeri FM, Lenze U, Wellauer V, von Eisenhart-Rothe R, Leunig M. Clinical improvement and satisfaction after total joint replacement: a prospective 12-month evaluation on the patients’ perspective. Qual Life Res. Netherlands; 2015;24: 2917–2925. doi: 10.1007/s11136-015-1042-3 26068733 10.1007/s11136-015-1042-3

[3] Roth P Von, Abdel MP, Wauer F, Winkler T, Wassilew G, Diederichs G, et al. Significant muscle damage after multiple revision total hip replacements through the direct lateral approach. 2014; 1618–1622. doi: 10.1302/0301-620X.96B12.34256 10.1302/0301-620X.96B12.34256 25452363

[4] Unis DB, Hawkins EJ, Alapatt MF, Benitez CL. Postoperative changes in the tensor fascia lata muscle after using the modified anterolateral approach for total hip arthroplasty. J Arthroplasty. United States; 2013;28: 663–665. doi: 10.1016/j.arth.2012.06.032 23253300 10.1016/j.arth.2012.06.032

[5] Gladstone JN, Bishop JY, Lo IKY, Flatow EL. American Journal of Sports Fatty Infiltration and Atrophy of the Rotator Cuff Do Not Improve After Rotator Cuff Repair and. Sport Med. 2007; 719–728. doi: 10.1177/0363546506297539 17337727 10.1177/0363546506297539

[6] Goodpaster BH, Kelley DE, Thaete FL, He J, Ross R. Skeletal muscle attenuation determined by computed tomography is associated with skeletal muscle lipid content. J Appl Physiol. 2000;89: 104–110. doi: 10.1152/jappl.2000.89.1.104 10904041 10.1152/jappl.2000.89.1.104

[7] Kiyoshige Y, Watanabe E. Fatty degeneration of gluteus minimus muscle as a predictor of falls. Arch Gerontol Geriatr. Elsevier Ireland Ltd; 2015;60: 59–61. doi: 10.1016/j.archger.2014.07.013 25440137 10.1016/j.archger.2014.07.013

[8] Daguet E, Jolivet E, Bousson V, Coutron C, Dahmen N, Bergot C, et al Fat Content of Hip Muscles: An Anteroposterior Gradient. J Bone Jt Surg. 2011;93–A: 1897–1905. 10.2106/JBJS.J.00509 22012527

[9] Bergmann G, Bergmann G, Deuretzabacher G, Deuretzabacher G, Heller M, Heller M, et al Hip forces and gait patterns from rountine activities. J Biomech. 2001;34: 859–871. http://dx.doi.org/10.1016/S0021-9290(01)00040-9 11410170 10.1016/s0021-9290(01)00040-9

[10] Bergmann G, Graichen F, Rohlmann A. Hip joint loading during walking and running, measured in two patients. J Biomech. 1993;26: 969–90. 8349721 10.1016/0021-9290(93)90058-m

[11] Bergmann G, Bender A, Dymke J, Duda G, Damm P. Standardized loads acting in hip implants. PLoS One. 2016;11: 1–23. doi: 10.1371/journal.pone.0155612 27195789 10.1371/journal.pone.0155612 PMC4873223

[12] Anderson FC, Pandy MG. Individual muscle contributions to support in normal walking. Gait Posture. 2003;17: 159–169. doi: 10.1016/s0966-6362(02)00073-5 12633777 10.1016/s0966-6362(02)00073-5

[13] Boren K, Conrey C, Le Coguic J, Paprocki L, Voight M, Robinson TK. ELECTROMYOGRAPHIC ANALYSIS OF GLUTEUS MEDIUS AND GLUTEUS MAXIMUS DURING REHABILITATION EXERCISES. International Journal of Sports Physical Therapy. Indianapolis, Indiana; 2011 pp. 206–223. 22034614 PMC3201064

[14] Correa TA, Crossley KM, Kim HJ, Pandy MG. Contributions of individual muscles to hip joint contact force in normal walking. J Biomech. Elsevier; 2010;43: 1618–1622. Available: http://www.ncbi.nlm.nih.gov/pubmed/20176362 doi: 10.1016/j.jbiomech.2010.02.008 20176362 10.1016/j.jbiomech.2010.02.008

[15] Gottschall JS, Okita N, Sheehan RC. Muscle activity patterns of the tensor fascia latae and adductor longus for ramp and stair walking. J Electromyogr Kinesiol. England; 2012;22: 67–73. doi: 10.1016/j.jelekin.2011.10.003 22074734 10.1016/j.jelekin.2011.10.003

[16] Heller MO, Schröder JH, Matziolis G, Sharenkov A, Taylor WR, Perka C, et al Muskuloskeletale belastungsanalysen. Biomechanische erklärung klinischer resultate—Und mehr? Orthopade. 2007;36: 188–194. doi: 10.1007/s00132-007-1054-y 17333071 10.1007/s00132-007-1054-y

[17] LIN H-C, LU T-W, HSU H-C. THREE-DIMENSIONAL ANALYSIS OF KINEMATIC AND KINETIC COORDINATION OF THE LOWER LIMB JOINTS DURING STAIR ASCENT AND DESCENT. Biomed Eng Appl Basis Commun. 2004;16: 101–108. doi: 10.4015/S1016237204000153

[18] Semciw AI, Green RA, Murley GS, Pizzari T. Gluteus minimus: an intramuscular EMG investigation of anterior and posterior segments during gait. Gait Posture. England; 2014;39: 822–826. doi: 10.1016/j.gaitpost.2013.11.008 24314814 10.1016/j.gaitpost.2013.11.008

[19] Arokoski MH, Arokoski JPA, Haara M, Kankaanpaa M, Vesterinen M, Niemitukia LH, et al Hip muscle strength and muscle cross sectional area in men with and without hip osteoarthritis. J Rheumatol. Canada; 2002;29: 2185–2195. 12375331

[20] Kienapfel H, Becker a. AE-Manual der Endoprothetik. AE-Manual der Endoprothetik. 2012; 419–440. doi: 10.1007/978-3-642-14646-6

[21] Putz C, Wolf SI, Geisbüsch A, Niklasch M, Döderlein L, Dreher T. Femoral derotation osteotomy in adults with cerebral palsy. Gait Posture. Elsevier B.V.; 2016;49: 290–296. doi: 10.1016/j.gaitpost.2016.06.034 27475618 10.1016/j.gaitpost.2016.06.034

[22] Petis S, Howard JL, Lanting BL, Vasarhelyi EM. Surgical approach in primary total hip arthroplasty: anatomy, technique and clinical outcomes. Can J Surg. 2015;58: 128–39. doi: 10.1503/cjs.007214 25799249 10.1503/cjs.007214 PMC4373995

[23] Muller M, Tohtz S, Springer I, Dewey M, Perka C. Randomized controlled trial of abductor muscle damage in relation to the surgical approach for primary total hip replacement: minimally invasive anterolateral versus modified direct lateral approach. Arch Orthop Trauma Surg. Germany; 2011;131: 179–189. doi: 10.1007/s00402-010-1117-0 20490520 10.1007/s00402-010-1117-0

[24] Masonis JL, Bourne RB. Surgical approach, abductor function, and total hip arthroplasty dislocation. Clin Orthop Relat Res. United States; 2002; 46–53. 12461355 10.1097/00003086-200212000-00006

[25] Springer I, Müller M, Hamm B, Dewey M. Intra- and interobserver variability of magnetic resonance imaging for quantitative assessment of abductor and external rotator muscle changes after total hip arthroplasty. Eur J Radiol. Elsevier Ireland Ltd; 2012;81: 928–933. doi: 10.1016/j.ejrad.2011.01.113 21354740 10.1016/j.ejrad.2011.01.113

[26] Heller MO, Bergmann G, Deuretzbacher G, Dürselen L, Pohl M, Claes L, et al Musculo-skeletal loading conditions at the hip during walking and stair climbing. J Biomech. 2001;34: 883–893. Available: http://www.sciencedirect.com/science/article/B6T82-437XPFY-6/2/2efde4e14ad48dd5b0864669b830fa6e 11410172 10.1016/s0021-9290(01)00039-2

[27] Colgan G, Walsh M, Bennett D, Rice J, O’Brien T. Gait analysis and hip extensor function early post total hip replacement. J Orthop. India; 2016;13: 171–176. doi: 10.1016/j.jor.2016.03.005 27408491 10.1016/j.jor.2016.03.005 PMC4919282

[28] Damm P, Dymke J, Ackermann R, Bender A, Graichen F, Halder A, et al Friction in total hip joint prosthesis measured in vivo during walking. PLoS One. 2013;8: 1–8. doi: 10.1371/journal.pone.0078373 24260114 10.1371/journal.pone.0078373 PMC3832636

[29] Engelken F, Wassilew GI, Köhlitz T, Brockhaus S, Hamm B, Perka C, et al Assessment of Fatty Degeneration of the Gluteal Muscles in Patients With THA Using MRI: Reliability and Accuracy of the Goutallier and Quartile Classi fi cation Systems. J Arthroplasty. Elsevier Inc.; 2014;29: 149–153. doi: 10.1016/j.arth.2013.04.045 23743509 10.1016/j.arth.2013.04.045

[30] Adolphson P, von Sivers K, Dalen N, Jonsson U, Dahlborn M. Bone and muscle mass after hip arthroplasty. A quantitative computed tomography study in 20 arthrosis cases. Acta Orthop Scand. England; 1993;64: 181–184. 8498182 10.3109/17453679308994566

[31] Rasch A, Byström AH, Dalén N, Martinez-Carranza N, Berg HE. Persisting muscle atrophy two years after replacement of the hip. J Bone &amp; Jt Surgery, Br Vol. 2009;91–B: 583 LP–588. 10.1302/0301-620X.91B5.21477 19407289

[32] Suetta C, Aagaard P, Rosted A, Jakobsen AK, Duus B, Kjaer M, et al Training-induced changes in muscle CSA, muscle strength, EMG, and rate of force development in elderly subjects after long-term unilateral disuse. J Appl Physiol. United States; 2004;97: 1954–1961. doi: 10.1152/japplphysiol.01307.2003 15247162 10.1152/japplphysiol.01307.2003

[33] Uemura K, Takao M, Sakai T. Volume Increases of the Gluteus Maximus, Gluteus Medius, and Thigh Muscles After Hip Arthroplasty. J Arthroplasty. Elsevier Ltd; 2016;31: 906–912.e1. doi: 10.1016/j.arth.2015.10.036 26652475 10.1016/j.arth.2015.10.036

[34] Muller M, Tohtz S, Winkler T, Dewey M, Springer I, Perka C. MRI findings of gluteus minimus muscle damage in primary total hip arthroplasty and the influence on clinical outcome. Arch Orthop Trauma Surg. Germany; 2010;130: 927–935. doi: 10.1007/s00402-010-1085-4 20221834 10.1007/s00402-010-1085-4

[35] Rodríguez-Roiz JM, Bori G, Tomas X, Fernández-Valencia JA, García-Díez AI, Pomés J, et al Hypertrophy of the tensor fascia lata muscle as a complication of total hip arthroplasty. Eur J Orthop Surg Traumatol. 2017;27: 255–259. doi: 10.1007/s00590-016-1854-z 27644425 10.1007/s00590-016-1854-z

[36] Sutter R, Kalberer F, Binkert CA, Graf N, Pfirrmann CWA, Gutzeit A. Abductor tendon tears are associated with hypertrophy of the tensor fasciae latae muscle. Skeletal Radiol. 2013;42: 627–633. doi: 10.1007/s00256-012-1514-2 22940837 10.1007/s00256-012-1514-2

[37] Mundermann A, Dyrby CO, Andriacchi TP. Secondary gait changes in patients with medial compartment knee osteoarthritis: increased load at the ankle, knee, and hip during walking. Arthritis Rheum. United States; 2005;52: 2835–2844. doi: 10.1002/art.21262 16145666 10.1002/art.21262

[38] Chang A, Hayes K, Dunlop D, Song J, Hurwitz D, Cahue S, et al Hip abduction moment and protection against medial tibiofemoral osteoarthritis progression. Arthritis Rheum. United States; 2005;52: 3515–3519. doi: 10.1002/art.21406 16255022 10.1002/art.21406

[39] Schwachmeyer V, Damm P, Bender A, Dymke J, Graichen F, Bergmann G. In vivo hip joint loading during post-operative physiotherapeutic exercises. PLoS One. 2013;8: e77807 doi: 10.1371/journal.pone.0077807 24204977 10.1371/journal.pone.0077807 PMC3812157

[40] Rahemi H, Nigam N, Wakeling JM. The effect of intramuscular fat on skeletal muscle mechanics: implications for the elderly and obese. J R Soc Interface. 2015;12: 20150365 doi: 10.1098/rsif.2015.0365 26156300 10.1098/rsif.2015.0365 PMC4535407

[41] Eng JJ, Chu KS. Reliability and comparison of weight-bearing ability during standing tasks for individuals with chronic stroke. Arch Phys Med Rehabil. 2002;83: 1138–1144. doi: 10.1053/apmr.2002.33644 12161837 10.1053/apmr.2002.33644 PMC3501528

[42] Shakoor N, Hurwitz DE, Block JA, Shott S, Case JP. Asymmetric knee loading in advanced unilateral hip osteoarthritis. Arthritis Rheum. 2003;48: 1556–1561. doi: 10.1002/art.11034 12794823 10.1002/art.11034

